# Artemisinins: their growing importance in medicine

**DOI:** 10.1016/j.tips.2008.07.004

**Published:** 2008-10

**Authors:** Sanjeev Krishna, Leyla Bustamante, Richard K. Haynes, Henry M. Staines

**Affiliations:** 1 Centre for Infection, Division of Cellular and Molecular Medicine, St. George's, University of London, Cranmer Terrace, London, SW17 0RE, UK; 2 Department of Chemistry, Open Laboratory of Chemical Biology, Institute of Molecular Technology for Drug Discovery and Synthesis, The Hong Kong University of Science and Technology, Clear Water Bay, Kowloon, Hong Kong, PR China

## Abstract

Artemisinins are derived from extracts of sweet wormwood (*Artemisia annua*) and are well established for the treatment of malaria, including highly drug-resistant strains. Their efficacy also extends to phylogenetically unrelated parasitic infections such as schistosomiasis. More recently, they have also shown potent and broad anticancer properties in cell lines and animal models. In this review, we discuss recent advances in defining the role of artemisinins in medicine, with particular focus on their controversial mechanisms of action. This safe and cheap drug class that saves lives at risk from malaria can also have important potential in oncology.

## Introduction

**Figure 1:**
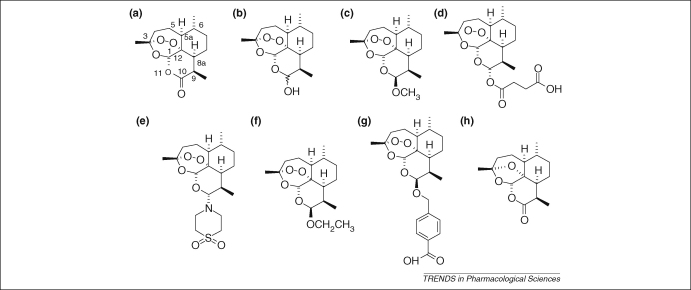
Chemical structures of artemisinins. Artemisinin **(a)** isolated in crystalline form in 1973 from *Artemisia annua* and derivatives dihydroartemisinin (DHA) **(b)**, artemether **(c)**, artesunate **(d)** and arteether **(f)** were first prepared by Chinese scientists in the 1970s [1]. Artemisone **(e)**, representative of a new class of artemisinin known as amino-artemisinins, is curative in clinical trials at one-third the dose regimen of artesunate. It is characterized by low toxicity [56]. Artelinate **(g)** was prepared at the Walter Reed Army Institute of Research (http://wrair-www.army.mil), but was withdrawn because of toxicity concerns [112]. Deoxyartemisinin **(h)**, lacking the peroxide bridge, is biologically inert.

The remarkable story of the discovery of artemisinin (Figure 1a) and establishment of its antimalarial activity by Chinese scientists represents one of the great discoveries in medicine in the latter half of the 20th century [1]. Through a collaborative effort, collectively referred to as ‘Project 523’, the Chinese prepared dihydroartemisinin (DHA; Figure 1b), artemether (Figure 1c) and artesunate (Figure 1d) in the 1970s. It is these derivatives [with others, including artemisone (Figure 1e), arteether (Figure 1f) and artelinic acid (Figure 1g), generically known as ‘artemisinins’] that are now making a crucial contribution to the management of malaria, one of our most important infections. The magnitude of the malaria problem is represented in the annual burden of 500 million cases. This fascinating class of drug, with structures so different from the classical quinoline antimalarials, is particularly valuable when used in combination with other antimalarials [2,3].

Artemisinins have also been submitted to studies aimed at exploring other uses for this drug class. Artemisinins are active against other parasite species *in vitro*, including protozoa that are phylogenetically unrelated to apicomplexan parasites such as the *Plasmodium* species that cause malaria. Artemisinins also act against metazoan parasites such as *Schistosoma* spp. Their anti-disease properties include potent anticancer activity in *in vitro* studies and in an *in vivo* model of colorectal cancer. Taken together with case reports describing benefits in diverse cancers, a recently published clinical trial of short-term use in lung cancer, their established record of safety in children and adults with malaria, and their permissive cost, there are compelling reasons to study their contribution to management of tumours that require adjuvant and neo-adjuvant therapies. This selective review focuses on rapidly advancing areas of artemisinin science and usage and illustrates why artemisinins have the potential to rival acetylsalicylic acid in the breadth of their anti-disease properties.

There is considerable debate regarding the mechanisms of antimalarial action of artemisinins. An endoperoxide bridge (Figure 1) lies at the heart of antiparasitic activity of artemisinins, although the chemical nature of the interaction between artemisinins (particularly the essential endoperoxide) and parasite target(s) is not well understood. The role of ferrous species in the antimalarial actions of artemisinins is also debated [4] because these cations can catalyse *in vitro* reactions of some artemisinins, including their decomposition in aqueous solutions.

One issue focuses further discussions: is there a single important target for artemisinins in *Plasmodium* spp. or are there multiple targets? Fully synthetic trioxolanes that contain an endoperoxide bridge but lack other features of artemisinins have increased complexity of the debate on mechanisms of action of artemisinins [5]. Many groups, including our own, have reviewed recent developments [6–9]. Clarifying mechanisms of action of artemisinins is important for understanding both how structurally related drugs, such as the fully synthetic trioxolanes, might work and the basis for the development of resistance by parasites to this class of antimalarial. Clearly, a structural appreciation of the putative targets should contribute to the design of derivatives that are not crippled by mutations in target, as exemplified by approaches used in the development of new dihydrofolate reductase inhibitors [10,11].

Rodent malarias are also useful models for understanding possible mechanisms of resistance to different classes of antimalarials [12,13]. Genetic analyses permitted by *Plasmodium chabaudi* infection in mice identified a locus linked to artemisinin resistance that is stable after mosquito passage [14,15]. Linkages to artemisinin resistance have been narrowed down to a de-ubiquitination enzyme (among others) that might function in the endoplasmic reticulum of parasites and be involved in the stress response. Other groups have established stable artemisinin-resistant strains, confirming that artemisinin resistance can develop through standard selection procedures rather than (unfortunately) being an extremely rare event and can also arise by more than one mechanism [16–18].

## Molecular targets of artemisinins

*Plasmodium falciparum* multiplies in red blood cells, and digestion of haemoglobin during its 48 h asexual life cycle is essential for parasite survival (Box 1). For many years, artemisinins have been proposed to act on parasite haemoglobin-digestion processes within the ‘food vacuole’ (Box 1, Figure Ib). Other studies have indicated that artemisinins could also target the parasite mitochondrion or the translationally controlled tumour protein (TCTP) and PfATP6, a parasite-encoded sarcoplasmic–endoplasmic reticulum calcium ATPase (SERCA). These hypotheses are discussed in more detail here.

### Haem pathway

Haemozoin is parasite pigment deposited within a food vacuole (Box 1) after digestion of haemoglobin. It has long been proposed as a target of artemisinins, although the plasmodial stages most susceptible to the activity of artemisinins are too young to manifest visible pigment (reviewed in Refs [19,20]). The endoperoxide bridge of artemisinins is proposed to be activated by ferrous iron to generate free radicals (of the oxy or C-centred variety) in *in vitro* experiments and, subsequently, to alkylate haem. As iron is the principal element deposited in haemozoin, digestion of haemoglobin by parasites is suggested to render them susceptible to killing by locally activated artemisinins.

However, several localization studies indicate that most artemisinin taken up into parasites is outside of their food vacuoles [21,22]. Some studies with fluorescent artemisinin derivatives show food vacuolar localization [23], perhaps representing trafficking of the fluorophore itself. This trafficking of a fully synthetic fluorescent antimalarial trioxolane might also explain differential localization results (one parasite with signal in the cytosol and the other in the food vacuole) observed for two parasites sharing the same erythrocyte [24]. Synthetic trioxolanes, such as OZ277, are more fragile than the semi-synthetic artemisinin derivatives when assayed in aqueous solutions [4,25,26], and they also seem to degrade easily within parasitized erythrocytes [27]. These properties might influence estimates of potency.

Further evidence for the irrelevance of parasite pigment in the action of artemisinins comes from their potent activity against non-pigment-producing apicomplexan parasites (see later). There is also divergence between some *in vitro* assays of haem alkylation by trioxolanes and natural and semi-synthetic artemisinins [25]. The correlation observed between antimalarial potencies of trioxolanes and their propensity to alkylate haem [25] is not observed for artemisinins, implying either that these classes of antimalarial might have different modes of action or that, indeed, the haem pathway might be irrelevant. The trioxolane OZ277 inhibits PfATP6 calcium ATPase activity when expressed in oocytes [24] at low (μM) concentrations. This might be owing to decomposition of the compound under the assay conditions or other aspects of the *in vitro* assay system. Study of more stable trioxolanes might resolve some of these issues. There is also correlation (*r*^2^ = 0.5, *n* = 38; *p* = 0.002) between parasiticidal activities of artesunate and OZ277 tested against field isolates, with no correlation between OZ277 and other classes of antimalarial such as quinolines [28]. This correlation might represent a general (non-target-specific) propensity of parasites to be susceptible to endoperoxides, but it is also consistent with the shared-target hypothesis for mechanisms of action, with PfATP6 being an example of such a target.

As a variant of the haem hypothesis, reaction with a histidine-rich protein of parasites (HRPII; Box 1) might also be involved in antimalarial activity [29] because HRPII aids digestion of haemoglobin. However, very little HRPII is secreted in early ring stages (Box 1), which are most susceptible to artemisinins [30,31].

Understanding interactions between haemoglobins and artemisinins is complicated by alterations in iron status associated with haemoglobinopathies. Higher concentrations of free iron in haemoglobin-E-containing and thalassaemic erythrocytes reduces parasiticidal potencies of artemisinins when assayed *in vitro* [32]. However, *in vivo* kinetic studies using bioassays of artesunate and its active metabolite, DHA, show approximately tenfold higher plasma concentrations in α-thalassaemic subjects when areas under the time–concentration curves were assessed [33], and the haemoglobin E trait might increase parasite clearance by artemisinins [34]. Despite these differences between *in vitro* activities of artemisinins related to the haemoglobin status of host erythrocytes, thalassaemia is not an influential co-variate in population pharmacokinetic analysis of rectal artesunate used to treat *Plasmodium vivax* or *P. falciparum* infections. Additionally, antimalarial activities of artemisinin against *P. falciparum* parasites cultured in the presence of carboxy-haemoglobin are significantly higher than in the presence of oxy-haemoglobin. This increase in artemisinin activity is unexpected if Fe^2+^ is important in activating artemisinins because carboxy-haemoglobin inhibits haem-Fe^2+^ reactivity, indicating that haemoglobin iron plays no part in activating artemisinin for antimalarial activity and that competitive degradation of the artemisinin by haemoglobin actually attenuates its antimalarial activity [35,36].

### PfATP6

The supportive arguments for PfATP6, the *P. falciparum* SERCA orthologue, as a target for artemisinins have been reviewed recently [9]. Evidence from transfection into parasites of DNA encoding PfATP6 that have altered sensitivity to some artemisinins will provide suitable genetic tests for the PfATP6 hypothesis (studies in progress), which has gained support from data from field isolates. An interesting study from French Guiana showed a clear association between mutation(s) in PfATP6 and decreased susceptibility to artemether, particularly with position 769 (Ser769Asn substitution) [37]. Parasites with Ser769Asn had a median IC_50_ value >20-times higher for artemether (indicating artemether resistance) compared with parasites without this mutation [9].

Detailed methodology for *in vitro* assays used in the earlier publication [37] is provided in a follow-up paper [38]. The lack of a laboratory-adapted line carrying the Ser769Asn mutation has been criticized, despite there being well-recognized ‘fitness-costs’ (i.e the ability of resistant parasites to persist in the absence of drug pressure) of some resistance mutations for cultured parasites, as shown for mutations in the *P. falciparum* multidrug resistance gene 1 (*pfmdr1*) [38–40]. Laboratory-derived transfectants carrying the Ser769Asn mutation will clarify its role in artemisinin resistance, especially when combined with *ex vivo* assays of susceptibility to artemisinins with the *Xenopus* oocyte model. An African isolate carrying the Ser769Asn mutation was still susceptible to DHA, and data for susceptibility to artemether were not reported (Table 1). These observations indicate that different artemisinin derivatives give rise to different inhibitory profiles when they encounter PfATP6 with a particular single-site polymorphism [41], as discussed elsewhere [42]. Structural modelling of the Ser769Asn mutation has proved difficult because the region containing this mutation has relatively low similarity to a mammalian SERCA (compared with other functionally conserved regions), a crystal structure of which is available [43]. This region is not related to the thapsigargin-binding site of mammalian SERCAs, which, in PfATP6, has also been hypothesized to accommodate artemisinins on the basis of mutational studies after expression in oocytes [44].

Mutation elsewhere in field isolates (position 243 in PfATP6) decreases susceptibility to DHA, although data are only available from two isolates [41]. Monitoring of polymorphisms in PfATP6 (and indeed other transporter sequences) and relating the findings to phenotypes by assessing susceptibility to artemisinins is likely to be highly relevant to the objective of detecting early signs of artemisinin resistance (Table 1). For example, increased copy number of the multidrug resistance gene *pfmdr1* modulates susceptibility of parasites to artemisinins *in vitro*, although the clinical relevance of this observation is not established [45].

### Other targets

Recent studies with Baker's yeast indicate that mitochondrial membrane potential can be disrupted by artemisinin when grown in nonfermentable conditions (i.e*.* when carbon sources such as glycerol or ethanol are not metabolized by glycolysis) [46]. However, the relevance of these observations to antimalarial activity of artemisinins is unclear because other experiments indicate that higher concentrations (mM) of artemisinins are necessary to trigger resistance responses to artemisinins in yeast [47]. Additionally, the new clinically tested artemisinin derivative artemisone has no effect on mitochondrial membrane potential, reactive oxygen species levels or inhibition of the respiratory chain in neuronal cell lines [48].

The TCTP orthologue of *P. falciparum* was identified some years ago as a protein alkylated by radiolabelled artemisinin. There is no new evidence that supports the idea of TCTP as a target for artemisinins. Field isolates that have variable sensitivities to artemether are not associated with sequence polymorphisms in TCTP [37]. Neither do studies with animal models of artemisinin-resistant parasites support involvement of TCTP as a target [15].

## Properties of artemisinins

### Antimalarial activity of artemisinins – clinical applications

Using artesunate to treat severe malaria in adults has been emphasized in recent publications [49]. Parenteral artesunate (including intramuscular artesunate [50]) is easier to administer and is associated with fewer adverse effects (e.g. hypoglycaemia) when compared with quinine [51], the only other drug used in severe malaria. Mortality in adults is also lower with artesunate than with quinine. Intrarectal treatment with artesunate of children or adults who cannot take medicines by mouth and suffer from symptoms of malaria away from healthcare facilities has also been studied in large scale (Phase IV) studies that will be reported soon. Both safety and efficacy have been established in smaller studies [52,53]. However, a child treated with very high rectal doses of artesunate (88 mg kg^−1^ in total compared with a recommended 10–20 mg kg^−1^) recently died because of probable toxicity [54].

Curiously, oral artemether and DHA are more commonly used in fixed-dose formulations rather than artesunate. Artesunate might have more favourable properties, both in terms of stability and ease of co-formulation when compared with DHA, and in terms of adverse effects in animal models when compared with artemether [55]. Newer semi-synthetic artemisinin derivatives such as artemisone (Figure 1e) preserve safety but enhance efficacy and should be studied for performance against models of artemisinin resistance [56].

### Activity against Toxoplasma gondii and other pathogenic apicomplexan parasites

Studying the susceptibility of non-plasmodial apicomplexans to artemisinins affords new therapeutic opportunities and provides new mechanistic insights. If organisms within the crown eukaryotic group are susceptible to artemisinins, then the simplest mechanistic interpretation is that they function in a similar way against these phylogenetically related organisms. For example, *Toxoplasma gondii* is a somewhat more tractable parasite than *Plasmodium* spp., particularly for studies using genetic manipulations or imaging technologies. Early work showed toxoplasma to be susceptible to artemisinins, albeit requiring concentrations within the micromolar range to kill parasites (online supplementary Table S1). Now studies show that *T. gondii* can be killed by nanomolar concentrations of artemisone in *in vitro* models and that TgSERCA (the PfATP6 orthologue) is susceptible to inhibition by artemisinin when expressed in yeast [57]. Furthermore, artemisinins trigger disturbances of calcium metabolism in parasites that have functional consequences on invasion machinery, and these might differ if parasites are cultured within host cells or as free living organisms [58]. These findings independently support the hypothesis that parasite SERCAs are targets for artemisinins (both *in vivo* and after heterologous expression). They also indicate that a glutamic acid residue predicted in transmembrane segment 3 of TgSERCA is permissive for artemisinin susceptibility [44], consistent with the suggestion made here that other key residues in TgSERCA might modulate artemisinin susceptibility.

Babesia species are tick-borne intraerythrocytic parasites that can infect humans in addition to a variety of domestic animals, depending on the species of parasite. Unlike plasmodial infections, babesia do not generate a parasitophorous vacuole and do not digest haemoglobin to make haemozoin [59]. Yet, some species are also susceptible to killing by artemisinins (online supplementary Table S1), once again making the haemoglobin digestion pathway a less compelling one for their mechanisms of action. Other related parasites have variable susceptibilities to artemisinins (online supplementary Table S1). These studies also establish that neither haemozoin nor haemoglobin is crucial to antiparasitic activity of artemisinins. It will be of interest to test the SERCA hypothesis for the mechanism of action of artemisinins in these related pathogenic parasites.

### Activity against other protozoan and metazoan parasites

Artemisinins are also active against phylogenetically unrelated parasites, such as the single-celled kinetoplastids and metazoan helminths (online supplementary Table S2; efficacy against *Schistosoma* spp. is reviewed elsewhere [60]). Both salivarian (African) and stercorarian (American) trypanosomes can be killed by micromolar concentrations of artemisinins, indicating that artemisinins can be used as leads on which to optimize more potent derivatives [61]. *Leishmania* spp. are also killed by micromolar concentrations of artemisinins (online supplementary Table S2). As these infections are usually neglected in drug development portfolios, it would be regrettable if promising *in vitro* activities are not examined more thoroughly in relevant *in vivo* models perhaps used in combination with current therapies.

For metazoan infections, particularly *Schistosoma* spp., artemether and artesunate have shown useful activities in human studies and in models of infection [60,62]. First identified in Chinese studies [63], these observations have been extended to African infections. The limited portfolio of active trematocidal compounds reinforces the potential for artemisinins in the treatment of *Schistosoma mansoni* and *Schistosoma haematobium*.

### Antitumour properties of artemisinins

Since the late 1980s, anticancer properties of artemisinins have been assayed *in vitro* (online supplementary Table S3). After more detailed studies, artemisinins such as artesunate were found to be active against a variety of unrelated tumour cells lines, from the most common types such as colon, breast and lung cancers to leukaemias and pancreatic cancer [64,65]. Studies have also identified potential general mechanisms such as normalization of the upregulated Wnt/β-catenin pathway in colorectal cancer [66]. Other pathways for anticancer activity include inhibition of enhanced angiogenesis associated with tumours [67–77]. Artemisinins inhibit proliferation, migration and tube formation of human umbilical vein endothelial cells (HUVEC), inhibit vascular endothelial growth factor (VEGF) binding to surface receptors on HUVEC and reduce expression of VEGF receptors Flt-1 and KDR/flk-1 on HUVECs [74,75,77]. In cancer cells, artemisinins reduce expression of the VEGF receptor KDR/flk-1 in tumour and endothelial cells and slow growth of human ovarian cancer HO-8910 xenografts in nude mice [67–69,75,77]. HUVEC apoptosis by artesunate is associated with downregulation of Bcl-2 (B-cell leukemia/lymphoma 2) and upregulation of BAX (Bcl-2-associated X protein) [78].

mRNA expression of 30 out of 90 angiogenesis-related genes correlated significantly with the cellular response to artemisinins [70]. In this microarray panel, there were many fundamental angiogenic regulators encoded by genes such as *VEGFC*, fibroblast growth factor-2 (*FGF2*), matrix metalloproteinase-9 (*MMP9*), thrombospondin-1 (*THBS1*) and hypoxia-inducing factor α (*HIF1A*). The fact that sensitivity and resistance of tumour cells can be predicted by mRNA expression levels of angiogenesis-related genes indicates that artemisinins reveal their antitumour effects, at least in part, by inhibition of tumour angiogenesis. Overexpression of enzymes associated with modulation of oxidative stress such as glutamylcysteine synthetase, glutathione S-transferases and the endothelial growth factor receptor reduce susceptibility of tumour cells to artemisinins [79,80]. Importantly, overexpression of genes encoding transporters that mediate drug resistance (e.g. multidrug resistance gene 1, multidrug resistance associated protein 1 and breast cancer resistance protein), dihydrofolate reductase and ribonucleotide reductase, which also confer resistance to established antitumour drugs, do not affect susceptibility, indicating that artemisinins function in different ways to classical cancer chemotherapeutic agents. These *in vitro* studies have also shown that for some cancer lines, delivery of iron, for example by the use of holotransferrin, enhances the anticancer properties of artemisinins [65,81–87].

Should artemisinins remain relegated to the large category of compounds that have interesting *in vitro* properties against cancers but have not been studied sufficiently to warrant more extensive clinical studies? Probably not, for many reasons. First, artesunate is a cheap, safe, easily administered and orally bioavailable compound that acts at targets different to those of many current cancer chemotherapeutic agents and is unlikely to interact adversely with existing anticancer interventions (P. Folb, personal communication). Second, study of an animal model carrying a human colorectal cancer cell line confirms that artesunate has independent antitumour activity and can shrink primary tumours and reduce the risk of hepatic metastases developing [66]. Additionally, human studies of individual cases [88,89], in addition to a recently published Phase II study of lung cancer [90], support rapid implementation of studies of artesunate as a primary or adjunct antitumour intervention, particularly for colorectal cancers and for leukaemia (as supported by results in online supplementary Table S3).

### Other potentially useful properties of artemisinin compounds

In *in vitro* studies, several groups have reported that artemisinins have antiviral properties. Artemisinins reduce replication rates of hepatitis B and C viruses [91,92], a range of human herpes viruses [93–95], HIV-1 [96], influenza virus A [93,97] and a bovine viral diarrhoea virus [98] in the low micromolar range. Artesunate was also effective at reducing CMV (human herpes virus 5) copy number in an immunosuppressed 12-year-old child [99] and was used (100 mg per day, orally) for 30 days without attributable toxicity. Artemisinins also have some antifungal properties against *Pneumocystis carinii in vitro* [100,101], although artemether was not curative in two *in vivo* studies in immunosuppressed rats [102,103]. There are several other disease models, such as those for rheumatoid arthritis [104–106], nephritic syndrome [107], pancreatitis [108] and lupus nephritis [109,110], in which artemisinins have produced promising results. In the case of lupus nephritis, artemisinin has been used for three years in a human study, with positive effects on the disease state [111].

## Concluding remarks

Artemisinins are firmly established in combination therapies [2,3] to treat drug-resistant malaria. They are becoming established as anti-schistosomal agents. Their true potential now lies in broader anti-disease applications, particularly in addressing the difficult challenge posed by advanced cancers for which expensive treatments are providing, at best, incremental gains in outcome. Questions about dosing regimens, safety of long-term use and possible interactions (either positive or negative) with existing therapies and toxicities that might be related to the treatment of tumours should be answered by appropriate clinical studies as part of an urgent need to investigate drugs such as artesunate for oncological indications.

## Figures and Tables

**Figure I:**
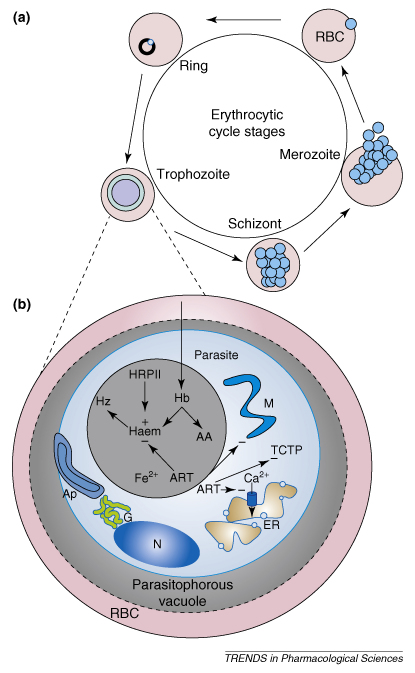
Diagram showing the complex life cycle of *Plasmodium falciparum.* Abbreviations: AA, amino acids; Ap, apicoplast; ART, artemisinins; DV, digestive vacuole; ER, endoplasmic reticulum; G, Golgi apparatus; Hb, haemoglobin; Hz, haemozoin; M, mitochondrion; N, nucleus; RBC, red blood cell; TCTP, translationally controlled tumour protein.

**Table 1:** Polymorphism in the *PfATPase6* gene and *in vitro* susceptibility to artemisinins of *Plasmodium falciparum*

Region	Non-synonymous nucleotide substitution	Amino acid substitution	Artemether IC_50_ median [range] (nM)	DHA IC_50_ median [range] (nM)	Artesunate IC_50_ median [range] (nM)	Refs
	Wild type	–	5.6 [1.3–55.8]	0.68 [0.1–31.8]	0.25 [0.17–18.4]	[37,41,45]
	5.46 [0.68–61.1]
Thailand	T266C	Ile89Thr	Not determined	Not determined	3.38 [0.81–29.9]	[45]
Africa	C727T	His243Tyr	Not determined	4.2; 6.4	Not determined	[41]
G2306A	Ser769Asn	Not determined	0.83	Not determined
Senegal	G1291A	Glu431Lys	Not determined	Not determined	20.8	[37]
G1291A and C1868A	Glu431Lys and Ala623Glu	Not determined	Not determined	44.7
French Guiana	G2306A	Ser769Asn	58.8 [38.2–100]	Not determined	Not determined	[37]
A1721C and G2306A	Gln574Pro and Ser769Asn	116.8	Not determined	Not determined

## Box 1 The intraerythrocytic parasite and proposed targets of artemisinins

Human malaria-causing parasites have complex life cycles requiring both mosquito vectors and human hosts with three cycles of asexual and one cycle of sexual reproduction. One of the asexual phases takes place within the red blood cells of its host (Figure Ia). Invasive forms, termed merozoites, enter the red blood cell and remain relatively metabolically inactive (compared with the later asexual stages of development) for 10–15 h (the ring stage). The parasite then undergoes a rapid phase of growth over the next 25 h (forming the trophozoite stage), during which time the parasite digests the majority of the haemoglobin of the host cell and grows to fill >50% of the volume of the host cell. Haemoglobin is digested within a food vacuole (Figure Ib), which results in the formation of haem. As the haem is formed, it associates via one of the peripheral carboxyl groups with the Fe^3+^ of an adjacent haem to form insoluble haemozoin. It has been proposed, although not proven, that this process is aided by a protein termed the histidine-rich protein II. At the end of the trophozoite stage the parasite divides several times (the schizont stage) before the host cell lyses (some 48 h after invasion) to release the newly formed meroziotes that continue the cycle.

Artemisinins, which might not require activation by Fe^2+^, have been proposed over several years to target several different pathways (Figure Ib), including the heam detoxification pathway, the mitochondrion, the TCTP and a Ca^2+^ pump localized to the endoplasmic reticulum (termed PfATP6).
