# Supplementary material for: Artemisinins: their growing importance in medicine
Source: Trends Pharmacol Sci. 2008 Oct;29(10):520–7. doi: 10.1016/j.tips.2008.07.004 (PMC2758403; doi:10.1016/j.tips.2008.07.004)
Supplement: Supplementary file 1 [file mmc1.doc]

Supplementary data

Artemisinins: their growing importance in medicine

Sanjeev Krishna^1^, Leyla Bustamante^1^, Richard K. Haynes^2^ and Henry M. Staines^1^

^1^ Centre for Infection, Division of Cellular and Molecular Medicine, St. George’s, University of London, Cranmer Terrace, London, SW17 0RE, UK

^2^ Department of Chemistry, Open Laboratory of Chemical Biology, Institute of Molecular Technology for Drug Discovery and Synthesis, The Hong Kong University of Science and Technology, Clear Water Bay, Kowloon, Hong Kong, PR China

*Corresponding author:* Krishna, S. (sgjf100@sgul.ac.uk).

Supplementary Table S1. Activity of clinically relevant artemisinins against Apicomplexan species excluding *Plasmodium* species.

| **Parasite/**  **species** | ***in vitro* or**  **animal**  **species** | **Derivative*** | **Effect of drug** | **Refs** |
| --- | --- | --- | --- | --- |
| ***Babesia*** |  |  |  |  |
| *microti* | Hamster | **4** | no effect, 160 mg/kg/d, 6d, *p.o.* | [1] |
| *microti* | Hamster | **7** | > 80% parasite suppression, 160 mg/kg/d, 6d, *p.o.* |  |
| *equi* | *in vitro* | **4** | IC_50_ = 0.26 µM | [2] |
| *caballi* | *in vitro* | **4** | IC_50_ = 0.47 µM |  |
| *equi* | Donkey | **6** | no effect, 2.5mg/kg, 4d, i.m. (unless buparvaquone is present) | [3] |
| *equi* | Donkey | **4** | increased survival, 5 mg/kg/d, 3d, i.m. |  |
| ***Haemoproteus*** |  |  |  |  |
| *tinnunculi* | Falcon | **3** | curative, 24 mg/3d, *p.o.* (in combination with 120 mg lumefantrine) | [4] |
| ***Toxoplasma*** |  |  |  |  |
| *gondii* | *in vitro* | **6** | no effect, 1.3 mM | [5] |
| *gondii* | Mouse | **6** | possible small effect, 200 mg/kg/d, 5d, s.c. |  |
| *gondii* | *in vitro* | **2**/**3**/**4**/**6** | > 98% inhibition at 4, 0.3, 3 and 0.3 µM, respectively | [6] |
| *gondii* | Mouse | **1** | no effect, 100 mg/kg/d, 5d, s.c. | [7] |
| *gondii* | *in vitro* | **6** | IC_50_ approximately 2 µM | [8] |
| *gondii* | Mouse | **3** | no curative effect, 100 mg/kg, 2/week, s.c. | [9] |
| *gondii* | *in vitro* | **1**/**2**/**3** | IC_50_ = 0.35, 0.35, and 0.13 µM, respectively | [10] |
| *gondii* | *in vitro* | **8** | no effect at 190 µM |  |
| *gondii* | *in vitro* | **2**/**4** | 70% and 40% parasite inhibition, respectively, 0.26 to 2 µM | [11] |
| *gondii* | Mouse | **2**/**4** (50/50) | 60% parasite reduction, 100 mg/kg/d, 5d, *p.o.* |  |
| *gondii* | Mouse | **3** | increased survival, 300 mg/kg/d, i.m. | [12] |
| *gondii* | *in vitro* | **1**/**3** | IC_50_ = 8 and 0.2 µM, respectively | [13] |
| *gondii* | *in vitro* | **1**/**2**/**3**/**4**/**5** | IC_50_ = 0.8, 0.4, 0.1, 0.1 and 0.03 µM, respectively | [14] |
| *gondii* | *in vitro* | **8** | IC_50_ = 10 µM |  |
| ***Eimeria*** |  |  |  |  |
| *tenella* | Chicken | **1** | reduced oocyst output, 17 ppm (in starter feed) or 2.5 mg/kg/d | [15, 16] |
| *acervalina* | Chicken | **1** | reduced oocyst output, 17 ppm (in starter feed) or 2.5 mg/kg/d |  |
| *maxima* | Chicken | **1** | no effect, 17 ppm (in starter feed) or 2.5 mg/kg/d |  |
| ***Cryptosporidium*** |  |  |  |  |
| *parvum* | Mouse | **1**/**3**/**6** | no curative effect, 200 mg/kg, s.c./i.r. | [17] |
| *parvum* | *in vitro* | **1** | ineffective at 7 µM | [18] |
| ***Neospora*** |  |  |  |  |
| *caninum* | *in vitro* | **1** | IC_50_ approximately 4 µM | [19] |
|  |  |  |  |  |

*Artemisinin **1,** DHA **2**, artemether **3**, artesunate **4**, artemisone **5**, arteether **6,** artelinate **7** and deoxyartemisinin **8**

1. Marley, S.E.*, et al.* (1997) Evaluation of selected antiprotozoal drugs in the *Babesia microti*-hamster model. *Antimicrob Agents Chemother* 41, 91-94

2. Nagai, A.*, et al.* (2003) Growth-inhibitory effects of artesunate, pyrimethamine, and pamaquine against *Babesia equi* and *Babesia caballi* in *in vitro* cultures. *Antimicrob Agents Chemother* 47, 800-803

3. Kumar, S.*, et al.* (2003) *In-vivo* therapeutic efficacy trial with artemisinin derivative, buparvaquone and imidocarb dipropionate against *Babesia equi* infection in donkeys. *J Vet Med Sci* 65, 1171-1177

4. Shaw, T., and Tarello, W. (2007) Treatment of *Haemoproteus tinnunculi* in falcons. *Vet Rec* 161, 360

5. Chang, H.R., and Pechere, J.C. (1988) Arteether, a qinghaosu derivative, in toxoplasmosis. *Trans R Soc Trop Med Hyg* 82, 867

6. Ke, O.Y.*, et al.* (1990) Inhibition of growth of *Toxoplasma gondii* by qinghaosu and derivatives. *Antimicrob Agents Chemother* 34, 1961-1965

7. Amato Neto, V.*, et al.* (1991) Eventual effect of artemisinine on the experimental infection of mice by *Toxoplasma gondii*. *Rev Soc Bras Med Trop* 24, 141-143

8. Holfels, E.*, et al.* (1994) *In vitro* effects of artemisinin ether, cycloguanil hydrochloride (alone and in combination with sulfadiazine), quinine sulfate, mefloquine, primaquine phosphate, trifluoperazine hydrochloride, and verapamil on *Toxoplasma gondii*. *Antimicrob Agents Chemother* 38, 1392-1396

9. Brun-Pascaud, M.*, et al.* (1996) Lack of activity of artemether for prophylaxis and treatment of *Toxoplasma gondii* and *Pneumocystis carinii* infections in rat. *Parasite* 3, 187-189

10. Berens, R.L.*, et al.* (1998) Selection and characterization of *Toxoplasma gondii* mutants resistant to artemisinin. *J Infect Dis* 177, 1128-1131

11. Sarciron, M.E.*, et al.* (2000) Effects of artesunate, dihydroartemisinin, and an artesunate-dihydroartemisinin combination against *Toxoplasma gondii*. *Am J Trop Med Hyg* 62, 73-76

12. Yao, J.M.*, et al.* (2003) Early treatment of *Toxoplasma gondii* infections with artemether in mice. *Zhongguo Ji Sheng Chong Xue Yu Ji Sheng Chong Bing Za Zhi* 21, 371

13. Jones-Brando, L.*, et al.* (2006) *In vitro* inhibition of *Toxoplasma gondii* by four new derivatives of artemisinin. *Antimicrob Agents Chemother* 50, 4206-4208

14. Nagamune, K.*, et al.* (2007) Artemisinin-resistant mutants of *Toxoplasma gondii* have altered calcium homeostasis. *Antimicrob Agents Chemother* 51, 3816-3823

15. Arab, H.A.*, et al.* (2006) Determination of artemisinin in *Artemisia sieberi* and anticoccidial effects of the plant extract in broiler chickens. *Trop Anim Health Prod* 38, 497-503

16. Allen, P.C.*, et al.* (1997) Effects of components of *Artemisia annua* on coccidia infections in chickens. *Poult Sci* 76, 1156-1163

17. Fayer, R., and Ellis, W. (1994) Qinghaosu (artemisinin) and derivatives fail to protect neonatal BALB/c mice against *Cryptosporidium parvum* (Cp) infection. *J Eukaryot Microbiol* 41, 41S

18. Giacometti, A.*, et al.* (1996) *In-vitro* activity of macrolides alone and in combination with artemisin, atovaquone, dapsone, minocycline or pyrimethamine against *Cryptosporidium parvum*. *J Antimicrob Chemother* 38, 399-408

19. Kim, J.T.*, et al.* (2002) *In vitro* antiprotozoal effects of artemisinin on *Neospora caninum*. *Vet Parasitol* 103, 53-63

Supplementary Table S2. Activity of clinically relevant artemisinins against non-Apicomplexan parasites excluding *Schistosoma* species.

| **Parasite/**  **species** | ***in vitro***  **or animal**  **species** | **Derivative*** | **Effect of drug** | **Refs** |
| --- | --- | --- | --- | --- |
| ***Naegleria*** |  |  |  |  |
| *fowleri* | *in vitro* | **1**/**2** | IC_50_ = 18 and 9 µM, respectively | [1] |
| *fowleri* | Mouse | **1**/**4**/**6** | not curative, up to 180 mg/kg/d, 5d, i.m. | [2, 3] |
| ***Acanthamoeba*** |  |  |  |  |
| *Group II/*  *polyphaga-like* | *in vitro* | **4** | IC_50_ approximately 130 µM | [4] |
| ***Gnathostoma*** |  |  |  |  |
| *spinigerum* | *in vitro* | **3** | no effect at 2 µM | [5] |
| ***Giardia*** |  |  |  |  |
| *lamblia* | *in vitro* | **2** | complete inhibition at 350 µM | [6] |
| ***Echinostoma*** |  |  |  |  |
| *caproni* | *in vitro* | **1**/**2**/**3**/**4**/**6** | killed 3/5, 5/5, 5/5, 5/5 and 5/5 worms, respectively, at 350, 4, 43, 3, 320 µM | [7] |
| *caproni* | Mouse | **1**/**3**/**4**/**6** | cured 3/4, 4/4, 4/4 and 3/4 mice, respectively, at single 1500, 1100,  700 and 1300 mg/kg doses, *p.o.* |  |
| ***Paragonimus*** |  |  |  |  |
| *westermani* | Dog | **3** | not curative at 25 mg/kg/d, 3d, *p.o.* | [8] |
| ***Clonorchis*** |  |  |  |  |
| *sinensis* | Rat | **3** | curative (100%) at 60 mg/kg/d, 7d, *p.o.* | [9] |
| *sinensis* | Human | **1** | no major effect at 1000 mg/d, 5d, *p.o.* | [10] |
| *sinensis* | Rat | **3**/**4** | curative (100%) at single 150 mg/kg doses, respectively, *p.o.* | [11, 12] |
| ***Opisthorchis*** |  |  |  |  |
| *viverrini* | Hamster | **3**/**4** | not curative (0/4 hamsters) and partially curative (2/4 hamsters) at single  400 mg/kg doses, *p.o.* | [11] |
| ***Fasciola*** |  |  |  |  |
| *hepatica* | *in vitro* | **2**/**3**/**4** | killed 4/4, 4/4, and 4/4 flukes, respectively, at 350, 340, and 260 µM | [13] |
| *hepatica* | Rat | **3**/**4** | cured 9/9 and 2/5 rats, respectively, at single 200 and 400 mg/kg doses, *p.o.* | [14] |
| *hepatica* | Sheep | **3** | significant egg and worm burden reductions at single 160 mg/kg dose, i.m. | [15] |
| ***Echinococcus*** |  |  |  |  |
| *multilocularis* | *in vitro* | **3** | no effect at 67 µM (even in presence of hemin) | [16] |
| ***Trypanosoma*** |  |  |  |  |
| *cruzi* | *in vitro* | **1**/**2**/**5** | IC_50_ = 13, 13 and 23 µM, respectively | [17] |
| *brucie rhod.* | *in vitro* | **1**/**2**/**5** | IC_50_ = 20, 25 and 23 µM, respectively |  |
| ***Leishmania*** |  |  |  |  |
| *major* | *in vitro* | **1**/**3** | IC_50_ = 0.75/30 (promastigotes/amastigotes) and 3 (amastigotes) µM, respectively | [18] |
| *major* | Mouse | **1**/**3**/**4** | reduced lesion size, 200 mg/kg/d, 5d, *p.o.* |  |
| *donovani* | *in vitro* | **1**/**2** | IC_50_ = 124 and 70 µM (promastigotes), respectively | [19] |
| *donovani* | *in vitro* | **2** | IC_90_ = 900 µM (promsatigotes) | [20] |
| *donovani* | Mouse | **2** | partially effective (80% reduction in parasite numbers) at 50 mg/kg/d, 14d, *p.o.* |  |
| *donovani* | *in vitro* | **1** | IC_50_ = 160/22 µM (promastigotes/amastigotes) | [21] |
| *donovani* | *in vitro* | **1**/**2**/**5** | IC_50_ = 31, 3 and 13 µM (promastigotes), respectively | [17] |

*Artemisinin **1,** DHA **2**, artemether **3**, artesunate **4**, artemisone **5**, arteether **6,** artelinate **7** and deoxyartemisinin **8**

1. Cooke, D.W.*, et al.* (1987) *In vitro* sensitivity of *Naegleria fowleri* to qinghaosu and dihydroqinghaosu. *J Parasitol* 73, 411-413

2. Gupta, S.*, et al.* (1998) Effect of alpha,beta-arteether against primary amoebic meningoencephalitis in Swiss mice. *Indian J Exp Biol* 36, 824-825

3. Gupta, S.*, et al.* (1995) *In vivo* study of artemisinin and its derivatives against primary amebic meningoencephalitis caused by *Naegleria fowleri*. *J Parasitol* 81, 1012-1013

4. Nacapunchai, D.*, et al.* (2002) *In vitro* effect of artesunate against Acanthamoeba spp. *Southeast Asian J Trop Med Public Health* 33 Suppl 3, 49-52

5. Sukontason, K.*, et al.* (2000) Lack of efficacy of quinine and artemether against advanced third-stage larvae of *Gnathostoma spinigerum in vitro*. *Southeast Asian J Trop Med Public Health* 31, 412-414

6. Tian, X.F.*, et al.* (2005) Effect of dihydroartemisinin on ultrastructure of *Giardia lamblia in vitro*. *Zhongguo Ji Sheng Chong Xue Yu Ji Sheng Chong Bing Za Zhi* 23, 292-295

7. Keiser, J.*, et al.* (2006) Trematocidal activity of praziquantel and artemisinin derivatives: *in vitro* and *in vivo* investigations with adult *Echinostoma caproni*. *Antimicrob Agents Chemother* 50, 803-805

8. Xue, J.*, et al.* (2008) Artemether and tribendimidine lack activity in experimental treatment of *Paragonimus westermani* in the dog. *Parasitol Res* 102, 537-540

9. Chen, R.X.*, et al.* (1983) Effects of qinghaosu and its derivatives on *C. sinensis* in rats. Yaoxue Tongbao 18. 410–11. *Yaoxue Tongbao* 18, 410–411 (Chemical abstracts, 100, 17251)

10. Tinga, N.*, et al.* (1999) Little effect of praziquantel or artemisinin on clonorchiasis in Northern Vietnam. A pilot study. *Trop Med Int Health* 4, 814-818

11. Keiser, J.*, et al.* (2006) Effect of artesunate and artemether against *Clonorchis sinensis* and *Opisthorchis viverrini* in rodent models. *Int J Antimicrob Agents* 28, 370-373

12. Xiao, S.H.*, et al.* (2008) Artemether, artesunate, praziquantel and tribendimidine administered singly at different dosages against *Clonorchis sinensis*: a comparative *in vivo* study. *Acta Trop* 106, 54-59

13. Keiser, J.*, et al.* (2006) Artesunate and artemether are effective fasciolicides in the rat model and *in vitro*. *J Antimicrob Chemother* 57, 1139-1145

14. Keiser, J.*, et al.* (2007) Activity of artemether and OZ78 against triclabendazole-resistant *Fasciola hepatica*. *Trans R Soc Trop Med Hyg* 101, 1219-1222

15. Keiser, J.*, et al.* (2008) Efficacy and safety of artemether against a natural *Fasciola hepatica* infection in sheep. *Parasitol Res*

16. Reuter, S.*, et al.* (2006) *In vitro* activities of itraconazole, methiazole, and nitazoxanide versus *Echinococcus multilocularis* larvae. *Antimicrob Agents Chemother* 50, 2966-2970

17. Mishina, Y.V.*, et al.* (2007) Artemisinins inhibit *Trypanosoma cruzi* and *Trypanosoma brucei rhodesiense in vitro* growth. *Antimicrob Agents Chemother* 51, 1852-1854

18. Yang, D.M., and Liew, F.Y. (1993) Effects of qinghaosu (artemisinin) and its derivatives on experimental cutaneous leishmaniasis. *Parasitology* 106, 7-11

19. Avery, M.A.*, et al.* (2003) Structure-activity relationships of the antimalarial agent artemisinin. 8. design, synthesis, and CoMFA studies toward the development of artemisinin-based drugs against leishmaniasis and malaria. *J Med Chem* 46, 4244-4258

20. Ma, Y.*, et al.* (2004) Activity of dihydroartemisinin against *Leishmania donovani* both *in vitro* and *vivo*. *Chin Med J (Engl)* 117, 1271-1273

21. Sen, R.*, et al.* (2007) Artemisinin triggers induction of cell-cycle arrest and apoptosis in *Leishmania donovani* promastigotes. *J Med Microbiol* 56, 1213-1218

Supplementary Table S3. Activity of clinically relevant artemisinins against cancer.

| **Cancer/**  **species** | ***in vitro***  **or animal**  **species** | **Derivative*** | **Effect of drug (and where applicable the cell line)** | **Refs** |
| --- | --- | --- | --- | --- |
| **Leukemia** |  |  |  |  |
| Human | *in vitro* | **4** | > 63% reduction in proliferation up to 1.3 mM (acute monocytic leukemia and Hodgkin’s disease patient blood samples) | [1] |
| Murine | *in vitro* | **1/8** | IC_50_ = 0.34 and > 38 µM, respectively (P-388) | [2] |
| Human | *in vitro* | **2** | IC_50_ = 2.6 µM (in presence holotransferrin), otherwise > 200 µM (Molt4) | [3-5] |
| Human | *in vitro* | **4** | IC_50_ = 0.14 µM (KG-1a) | [6] |
| Human | *in vitro* | **4** | IC_50_ = 1.1 µM (mean of 6 cell lines including K562, Molt4 and CCRF-CEM) | [7] |
| Human | *in vitro* | **1**/**4** | IC_50_ = 34 and 0.7 µM, respectively (CCRF-CEM) | [8] |
| Human | *in vitro* | **1**/**4** | IC_50_ = 12 and 1.1 µM, respectively (CCRF-CEM) | [9] |
| Human | *in vitro* | **2** | IC_50_ = 1.64 µM (Molt4) | [10] |
| Human | *in vitro* | **2** | IC_50_ = 13 µM (K562) | [11] |
| Human | *in vitro* | **1**/**2**/**4** | IC_50_ = 15, 1 and 1 µM, respectively (K562) | [12] |
| Human | *in vitro* | **2** | IC_50_ = 13 µM (K562) | [13] |
| Human | *in vitro* | **2** | IC_50_ = 2.4/0.7 µM (HL60/Jurkat) | [14] |
| Human | *in vitro* | **4** | MIC = 5/16/1/0.3 µM (Jurkat/Hut 78/Molt4/CCRF-CEM) | [15] |
| Human | *in vitro* | **4** | IC_50_ approximately 3 µM (U937) | [16] |
| Human | *in vitro* | **2** | IC_50_ between 0.25 and 8 µM (HL60) | [17] |
| Human | *in vitro* | **1** | IC_50_ >20 µM (HL60) | [18] |
| **Lung** |  |  |  |  |
| Human | *in vitro* | **1/8** | IC_50_ = 15 and > 38 µM, respectively (P-388) | [2] |
| Human | *in vitro* | **1**/**2**/**4** | IC_50_ = 34, 0.5 and 0.6 µM, respectively (GLC4) | [12] |
| Human | *in vitro* | **4** | IC_50_ = 26 µM (mean of 6 cell lines including A549) | [7] |
| Human | *in vitro* | **4** | IC_50_ = 0.002/0.024 µM (H69/resistant H69) | [19] |
| Human | *in vitro* | **2** | IC_50_ = 18 µM (PC-14) | [20] |
| Human | Human | **4** | partially effective (time to progression longer) at 120 mg/d, 8d, i.v. | [21] |
| Human | *in vitro* | **4** | IC_50_ = 1.2 to 70 µM (6 cell lines) | [22] |
| **Liver** |  |  |  |  |
| Murine | Mouse | **4** | partially effective (tumor inhibitory rates were 45 to 50%) at 300 mg/kg/d, 7d, i.g. (H22) | [23, 24] |
| Human | *in vitro* | **4** | IC_50_ = 5 µM (SMMC-7721) | [23] |
| Human | *in vitro* | **2** | IC_50_ = 246 µM (HepB3) | [25] |
| **Stomach** |  |  |  |  |
| Human | *in vitro* | **4** | IC_50_ = 0.6 µM (GXF 251L) | [22] |
| **Breast** |  |  |  |  |
| Murine | *in vitro* | **1**/**2**/**3**/**4**/**6**/**7** | IC_50_ = 30, 15, 15, 15, 15 and 15 µM, respectively (Ehrlich ascites) | [26] |
| Human | *in vitro* | **1/8** | IC_50_ > 35 and 38 µM , respectively (MCF7) | [2] |
| Human | *in vitro* | **4** | IC_50_ = 18 µM (mean of 8 cell lines including MCF7) | [7] |
| Human | *in vitro* | **2** | IC_50_ = 608 µM (MDA-MB-231) | [25] |
| Murine | Rat | **1** | partially effective (delayed tumour development) at 8 mg/kg/g, 280 d, *p.o.* | [27] |
| Human | *in vitro* | **4** | IC_50_ = 0.75/1.7 µM (MAXF-401NL/MCF7) | [22] |
| **Colon** |  |  |  |  |
| Human | *in vitro* | **1/8** | IC_50_ = 16 and > 38 µM (HT29) | [2] |
| Human | *in vitro* | **4** | IC_50_ = 2.1 µM (mean of 7 cell lines including HT29) | [7] |
| Human | *in vitro* | **4** | IC_50_ = 1.4/31 µM (HT29/HCT16) | [22] |
| Human | *in vitro* | **4** | IC_50_ = 20 µM (CLY) | [28] |
| Human | Mouse | **4** | partially effective (50% less tumour weight) at 300 mg/kg/3d, 21d, i.v. (CLY) |  |
| Human | *in vitro* | **4** | IC_50_ = 20/31/82 µM (CLY/Lovo/HT29) | [29] |
| **CNS** |  |  |  |  |
| Murine | *in vitro* | **1**/**2**/**3**/**4**/**6**/**7*/*8** | IC_50_ = 100/100, 0.5/0.8, 49/100, 0.5/0.5, 11/4, 69/66 and 100/100 µM, respectively (NG108-15/Nb2a) | [30] |
| Murine | *in vitro* | **1**/**2**/**3**/**6**/**7**/**8** | IC_50_ = 0.3, 6.4/1.6, 0.7/14, 0.3/4.3, 44.2 and >100/>100 µM, respectively (Nb2a/C6), effect enhanced by haemin | [31] |
| Human | *in vitro* | **1**/**4** | IC_50_ = 3.3 and 3.5 µM, respectively (U373) | [9] |
| Human | *in vitro* | **4** | IC_50_ = 17 µM (mean of 6 cell lines) | [7] |
| Human | *in vitro* | **4** | IC_50_ = 1.2/11 µM (CNXF 498NL/SF268) | [22] |
| Human | *in vitro* | **2** | IC_50_ < 140 microM (6 cell lines including U373MG) | [25] |
| Murine | *in vitro* | **2** | IC_50_ between 2 and 25 µM (C6), effect enhanced by Fe^2+^ | [32] |
| Murine | *in vitro* | **2** | IC_50_ = 23 µM (C6) | [33, 34] |
| **Head and Neck** |  |  |  |  |
| Human | *in vitro* | **4** | IC_50_ = 1.6 µM (HNXF S36L) | [22] |
| Human | Human | **3** | tumour static effect, 0.5 mg/kg/d, 29 d, *p.o.* | [35] |
| Human | *in vitro* | **1**/**2** | IC_50_ = 544 and 40 µM, respectively (YD-10B) | [36] |
| Canine | Dog | **2** | curative in 2/3 cases, 78 mM topically/d, 5d/week for 5 weeks | [37] |
| **Melanoma** |  |  |  |  |
| Human | *in vitro* | **4** | IC_50_ = 10 µM (mean of 7 cell lines) | [7] |
| Human | *in vitro* | **4** | IC_50_ = 2.2 to 72 µM (5 cell lines) | [22] |
| Human | Human | **4** | increased survival times at 100 mg/d, > 20 months, *p.o.* | [38] |
| **Ovarian** |  |  |  |  |
| Human | *in vitro* | **4** | IC_50_ = 14 µM (mean of 6 cell lines) | [7] |
| Human | *in vitro* | **4** | IC_50_ = 0.5 to 47 µM (3 cell lines) | [22] |
| Human | *in vitro* | **1**/**2/4** | IC_50_ = 50, 33 and 50 µM, respectively (HO-8910) | [39, 40] |
| Human | Mouse | **4** | partially effective (tumour growth decreased) at > 10 mg/kg/d, 15 d, s.c. (HO-8910) | [40, 41] |
| Human | *in vitro* | **1**/**3**/**4**/**6** | IC_50_ < 20 µM (OVCA-432 and SK-OV-3) | [42] |
| Human | *in vitro* | **2** | IC_50_ = 4 to 15 microM (10 cell lines) |  |
| Human | *in vitro* | **1**/**2**/**3**/**4** | IC_50_ = >500/343, 16/6.7, 54/39 and18/6.9 µM, respectively (A2780/OVCAR3) | [43] |
| Human | Mouse | **2** | partially effective (tumour growth decreased) at ≥ 10 mg/kg, 5d/week, 18 d, i.p. ( A2780/OVCAR3) |  |
| **Renal** |  |  |  |  |
| Human | *in vitro* | **4** | IC_50_ = 12 µM (mean of 6 cell lines) | [7] |
| Human | *in vitro* | **4** | IC_50_ = 1.8 to 53 µM (4 cell lines) | [22] |
| **Prostate** |  |  |  |  |
| Human | *in vitro* | **4** | IC_50_ = 11 µM (mean of 2 cell lines) | [7] |
| Human | *in vitro* | **4** | IC_50_ = 5.3 to 27 µM (4 cell lines) | [22] |
| Human | *in vitro* | **4** | Arrests differentiation and cell cycle at > 10 µM (PC-3) | [22] |
| **Pancreas** |  |  |  |  |
| Human | *in vitro* | **4** | IC_50_ = 1.7/124 µM (PANC1/PAXF 1657L) | [22] |
| **Endometrium** |  |  |  |  |
| Human | *in vitro* | **4** | IC_50_ = 2.1 µM (UXF 1138L) | [22] |
| **Bladder** |  |  |  |  |
| Human | *in vitro* | **4** | IC_50_ = 4.5/28 µM (T24/BXF 1218L) | [22] |
| **Pleural** |  |  |  |  |
| Human | *in vitro* | **4** | IC_50_ = 26 µM (PXF 1752L) | [22] |
| **Cervical** |  |  |  |  |
| Human | *in vitro* | **2** | IC_50_ = 669 µM (HeLa) | [25] |
| Human | *in vitro* | **1**/**2/4** | IC_50_ = 39, 16 and 39 µM , respectively (HeLa) | [39, 40] |
| Human | *in vitro* | **1**/**2**/**4** | IC_50_ > 50, = 8/12/7 and 5 µM, respectively (HeLa/SiHa/Caski) | [37] |
| **Uterus** |  |  |  |  |
| Human | *in vitro* | **1**/**2/4** | IC_50_ = 40, 25 and 40 µM, respectively (JAR) | [39, 40] |
| Human | *in vitro* | **1** | IC_50_ = 7 µM (JAR) | [44] |
| **Sarcoma** |  |  |  |  |
| Human | *in vitro* | **1**/**2** | IC_50_ = 15 and 9 µM, respectively (RD) | [39] |
| Murine | Rat | **2** | partially effective (tumour reduction with ferrous SO4), 2 mg/kg/d, 3d, then 5 mg/kg/d, 8d, *p.o.* | [45] |
| Human | *in vitro* | **4** | IC_50_ = 6 µM (KS-IMM) | [46] |
| Human | Mouse | **4** | partially effective (suppressed tumour development) at 167 mg/kg/d, 21d, *p.o.* | [46] |
| Murine | *in vitro* | **3/4** | IC_50_ > 27 and > 21 µM (WEHI-164) | [47, 48] |
|  |  |  |  |  |

*Artemisinin **1,** DHA **2**, artemether **3**, artesunate **4**, artemisone **5**, arteether **6,** artelinate **7** and deoxyartemisinin **8**

1. Shen, M.*, et al.* (1984) Immunosuppressive action of Qinghaosu. *Sci Sin* 27, 398-406

2. Zheng, G.Q. (1994) Cytotoxic terpenoids and flavonoids from *Artemisia annua*. *Planta Med* 60, 54-57

3. Lai, H., and Singh, N.P. (1995) Selective cancer cell cytotoxicity from exposure to dihydroartemisinin and holotransferrin. *Cancer Lett* 91, 41-46

4. Singh, N.P., and Lai, H.C. (2004) Artemisinin induces apoptosis in human cancer cells. *Anticancer Res* 24, 2277-2280

5. Singh, N.P., and Lai, H.C. (2005) Synergistic cytotoxicity of artemisinin and sodium butyrate on human cancer cells. *Anticancer Res* 25, 4325-4331

6. Efferth, T.*, et al.* (1996) Detection of apoptosis in KG-1a leukemic cells treated with investigational drugs. *Arzneimittelforschung* 46, 196-200

7. Efferth, T.*, et al.* (2001) The anti-malarial artesunate is also active against cancer. *Int J Oncol* 18, 767-773

8. Efferth, T.*, et al.* (2002) Activity of drugs from traditional Chinese medicine toward sensitive and MDR1- or MRP1-overexpressing multidrug-resistant human CCRF-CEM leukemia cells. *Blood Cells Mol Dis* 28, 160-168

9. Efferth, T.*, et al.* (2004) Enhancement of cytotoxicity of artemisinins toward cancer cells by ferrous iron. *Free Radic Biol Med* 37, 998-1009

10. Lai, H.*, et al.* (2005) Effects of artemisinin-tagged holotransferrin on cancer cells. *Life Sci* 76, 1267-1279

11. Lee, J.*, et al.* (2006) Dihydroartemisinin downregulates vascular endothelial growth factor expression and induces apoptosis in chronic myeloid leukemia K562 cells. *Cancer Chemother Pharmacol* 57, 213-220

12. Reungpatthanaphong, P., and Mankhetkorn, S. (2002) Modulation of multidrug resistance by artemisinin, artesunate and dihydroartemisinin in K562/adr and GLC4/adr resistant cell lines. *Biol Pharm Bull* 25, 1555-1561

13. Li, J., and Zhou, H.J. (2005) Dihydroartemisinin inhibits the expression of vascular endothelial growth factor in K562 cells. *Yao Xue Xue Bao* 40, 1041-1045

14. Mercer, A.E.*, et al.* (2007) Evidence for the involvement of carbon-centered radicals in the induction of apoptotic cell death by artemisinin compounds. *J Biol Chem* 282, 9372-9382

15. Efferth, T.*, et al.* (2007) Artesunate induces ROS-mediated apoptosis in doxorubicin-resistant T leukemia cells. *PLoS ONE* 2, e693

16. Zheng, Z.Y.*, et al.* (2007) Immune response of dendritic cells capturing antigens from apoptotic U937 cells induced by artesunate. *Zhongguo Shi Yan Xue Ye Xue Za Zhi* 15, 833-838

17. Zhou, H.J.*, et al.* (2008) Dihydroartemisinin induces apoptosis in human leukemia cells HL60 via downregulation of transferrin receptor expression. *Anticancer Drugs* 19, 247-255

18. Kim, S.H.*, et al.* (2008) Interferon-alpha enhances artemisinin-induced differentiation of HL-60 leukemia cells via a PKCalpha/ERK pathway. *Eur J Pharmacol* 587, 65-72

19. Sadava, D.*, et al.* (2002) Transferrin overcomes drug resistance to artemisinin in human small-cell lung carcinoma cells. *Cancer Lett* 179, 151-156

20. Mu, D.*, et al.* (2008) The role of calcium, P38 MAPK in dihydroartemisinin-induced apoptosis of lung cancer PC-14 cells. *Cancer Chemother Pharmacol* 61, 639-645

21. Zhang, Z.Y.*, et al.* (2008) Artesunate combined with vinorelbine plus cisplatin in treatment of advanced non-small cell lung cancer: A randomized controlled trial. *Zhong Xi Yi Jie He Xue Bao* 6, 134-138

22. Kelter, G.*, et al.* (2007) Role of transferrin receptor and the ABC transporters ABCB6 and ABCB7 for resistance and differentiation of tumor cells towards artesunate. *PLoS ONE* 2, e798

23. Wang, Q.*, et al.* (2001) Experimental studies of antitumor effect of artesunate on liver cancer. *Zhongguo Zhong Yao Za Zhi* 26, 707-708, 720

24. Wang, Q.*, et al.* (2002) The anticancer effect of artesunate and its mechanism. *Yao Xue Xue Bao* 37, 477-478

25. Kim, S.J.*, et al.* (2006) Dihydroartemisinin enhances radiosensitivity of human glioma cells *in vitro*. *J Cancer Res Clin Oncol* 132, 129-135

26. Woerdenbag, H.J.*, et al.* (1993) Cytotoxicity of artemisinin-related endoperoxides to Ehrlich ascites tumor cells. *J Nat Prod* 56, 849-856

27. Lai, H., and Singh, N.P. (2006) Oral artemisinin prevents and delays the development of 7,12-dimethylbenz[a]anthracene (DMBA)-induced breast cancer in the rat. *Cancer Lett* 231, 43-48

28. Li, L.N.*, et al.* (2007) Artesunate attenuates the growth of human colorectal carcinoma and inhibits hyperactive Wnt/beta-catenin pathway. *Int J Cancer* 121, 1360-1365

29. Li, L.N.*, et al.* (2008) Differential sensitivity of colorectal cancer cell lines to artesunate is associated with expression of beta-catenin and E-cadherin. *Eur J Pharmacol* 588, 1-8

30. Wesche, D.L.*, et al.* (1994) Neurotoxicity of artemisinin analogs *in vitro*. *Antimicrob Agents Chemother* 38, 1813-1819

31. Fishwick, J.*, et al.* (1995) The toxicity of artemisinin and related compounds on neuronal and glial cells in culture. *Chem Biol Interact* 96, 263-271

32. Huang, X.J.*, et al.* (2007) Dihydroartemisinin exerts cytotoxic effects and inhibits hypoxia inducible factor-1alpha activation in C6 glioma cells. *J Pharm Pharmacol* 59, 849-856

33. Huang, X.J.*, et al.* (2008) Dihydroartemisinin potentiates the cytotoxic effect of temozolomide in rat c6 glioma cells. *Pharmacology* 82, 1-9

34. Ma, Z.Q.*, et al.* (2007) Dihydroartemisinin inhibits proliferation and induces apoptosis of rat glioma C6 cells. *Zhejiang Da Xue Xue Bao Yi Xue Ban* 36, 267-272

35. Singh, N.P., and Panwar, V.K. (2006) Case report of a pituitary macroadenoma treated with artemether. *Integr Cancer Ther* 5, 391-394

36. Nam, W.*, et al.* (2007) Effects of artemisinin and its derivatives on growth inhibition and apoptosis of oral cancer cells. *Head Neck* 29, 335-340

37. Disbrow, G.L.*, et al.* (2005) Dihydroartemisinin is cytotoxic to papillomavirus-expressing epithelial cells *in vitro* and *in vivo*. *Cancer Res* 65, 10854-10861

38. Berger, T.G.*, et al.* (2005) Artesunate in the treatment of metastatic uveal melanoma--first experiences. *Oncol Rep* 14, 1599-1603

39. Chen, H.H.*, et al.* (2003) Inhibition of human cancer cell line growth and human umbilical vein endothelial cell angiogenesis by artemisinin derivatives in vitro. *Pharmacol Res* 48, 231-236

40. Chen, H.H.*, et al.* (2004) Inhibitory effects of artesunate on angiogenesis and on expressions of vascular endothelial growth factor and VEGF receptor KDR/flk-1. *Pharmacology* 71, 1-9

41. Chen, H.H., and Zhou, H.J. (2004) Inhibitory effects of artesunate on angiogenesis. *Yao Xue Xue Bao* 39, 29-33

42. Jiao, Y.*, et al.* (2007) Dihydroartemisinin is an inhibitor of ovarian cancer cell growth. *Acta Pharmacol Sin* 28, 1045-1056

43. Chen, T.*, et al.* (2008) Dihydroartemisinin induces apoptosis and sensitizes human ovarian cancer cells to carboplatin therapy. *J Cell Mol Med*

44. Nilkaeo, A.*, et al.* (2006) Induction of cell cycle arrest and apoptosis in JAR trophoblast by antimalarial drugs. *Biomed Res* 27, 131-137

45. Moore, J.C.*, et al.* (1995) Oral administration of dihydroartemisinin and ferrous sulfate retarded implanted fibrosarcoma growth in the rat. *Cancer Lett* 98, 83-87

46. Dell'Eva, R.*, et al.* (2004) Inhibition of angiogenesis *in vivo* and growth of Kaposi's sarcoma xenograft tumors by the anti-malarial artesunate. *Biochem Pharmacol* 68, 2359-2366

47. Cuzzocrea, S.*, et al.* (2005) Artemether: a new therapeutic strategy in experimental rheumatoid arthritis. *Immunopharmacol Immunotoxicol* 27, 615-630

48. Mirshafiey, A.*, et al.* (2006) Design of a new line in treatment of experimental rheumatoid arthritis by artesunate. *Immunopharmacol Immunotoxicol* 28, 397-410
